# Preparation and Thermal Conductivity Enhancement of Boron Nitride Nano-Material PiG Composite

**DOI:** 10.3390/nano13061106

**Published:** 2023-03-20

**Authors:** Zhenhua Chen, Qinhua Wei, Gao Tang, Hongsheng Shi, Laishun Qin

**Affiliations:** 1 College of Materials and Chemistry, China Jiliang University, Hangzhou 310018, China; 2 Xinjiang Technical Institute of Physics and Chemistry, Chinese Academy of Sciences, Urumqi 830011, China

**Keywords:** boron nitride, phosphor in glass, thermal conductivity enhancement

## Abstract

With the improvement of the conversion efficiency of LED chip and fluorescent material and the increasing demand for high-brightness light sources, LED technology has begun to move toward the direction of high-power. However, there is a huge problem that high-power LED must face with a large amount of heat generated by high power causing a high temperature thermal decay or even thermal quenching of the fluorescent material in the device, resulting in a reduction of the luminous efficiency, color coordinates, color rendering index, light uniformity, and service life of LED. In order to solve this problem, fluorescent materials with high thermal stability and better heat dissipation were prepared to enhance their performance in high-power LED environments. A variety of boron nitride nanomaterials were prepared by the solid phase-gas phase method. By adjusting the ratio of boric acid to urea in the raw material, different BN nanoparticles and nanosheets were obtained. Moreover, the control of catalyst amount and synthesis temperature can be used to synthesize boron nitride nanotubes with various morphologies. By adding different morphologies and quantities of BN material in PiG (phosphor in glass), the mechanical strength, heat dissipation, and luminescent properties of the sheet can be effectively controlled. PiG prepared by adding the right number of nanotubes and nanosheets has higher quantum efficiency and better heat dissipation after being excited by high power LED.

## 1. Introduction

When the heat conduction or heat dissipation performance of the fluorescent layer in the remote packaging structure is improved, it can play a better role in the high-power working environment. Therefore, in the previous work, we coated an appropriate GO/rGO film on the surface of PiG to improve its surface thermal conductivity and ultimately enhance its luminous performance in high-power LED.

However, the coating of GO/rGO film can only enhance the surface heat dissipation performance of PiG, while the heat inside the fluorescent layer still needs to be dissipated through its own conduction, so it will still accumulate a lot of heat inside, resulting in a decrease in luminous efficiency [1,2].

Therefore, it is of great significance to enhance the internal thermal conductivity of the fluorescent film. Song et al. mixed rGO and phosphor into PDMS to make a fluorescent film with internal graphene heat dissipation structure for remote packaging [3]. Anoop’s team coated graphene on the surface of phosphor particles and mixed it with organic materials for packaging, thus regulating the thermal stability of LED [4]. However, these films cannot be used in high-power excitation environments due to the defects of organic materials. Due to the poor high-temperature stability of graphene in the aerobic environment [5], it is easily oxidized and can even cause further fluorescence quenching in the preparation of fluorescent ceramic and fluorescent glass. Therefore, it is impossible to use the internal embedding or coating of graphene to enhance the internal thermal conductivity of PiG. It is necessary to find other more suitable materials or methods.

It is easy to associate graphene with another similar material, boron nitride. Boron nitride is composed of B and N, which are adjacent to C in the periodic table of chemical elements, so BN and C have many similarities in properties [6,7].

The structures of block, monolayer, and nanotube six-membered ring BN materials are similar to those of graphite, graphene, and carbon nanotubes, respectively [8]. Similar structures also bring similar physical and chemical properties, such as excellent thermal conductivity and mechanical properties. At the same time, BN also has many properties that carbon materials do not have, including good thermal stability, chemical stability and macroscopic transmittance to visible light [9,10,11]. Taken together, it can be found that boron nitride is very suitable as the internal heat transfer material of PiG and can also play a certain role in enhancing the mechanical properties of PiG [12,13].

There are few reports about BN reinforcing PiG, and the related research mainly focuses on the strengthening of boron nitride on ceramic matrix and polymer. Researchers have used boron nitride nanotube (BNNT) to strengthen alumina ceramics, hydroxyapatite artificial bone materials, and glass matrix [14,15,16,17], and filled polymers, such as polyethylene, with boron nitride nanosheet (BNNS) and boron nitride nanoparticle (BNNP) to enhance thermal conductivity [18,19]. These works have to some extent verified the feasibility of BN enhancing PiG.

In this work, boron nitride nanomaterials with different morphologies are prepared by adjusting the reaction composition, catalyst, and synthesis temperature. After that, different amounts and types of BN materials are added to PiG to explore their effects on the thermal conductivity and mechanical properties of phosphors. Finally, fluorescent films with strong thermal stability and mechanical properties are obtained and applied to the remote packaging of high-power LEDs.

## 2. Materials and Methods

### 2.1. Preparation of BN Nanoparticles and Nano-Sheets

Mix boric acid and urea in a certain proportion (1:2~16 molar ratio) at room temperature, put the powder into a covered crucible, and put it into a tubular atmosphere furnace. The temperature is raised to 950 °C under argon protection atmosphere, and then ammonia is introduced, before the temperature is raised to 1000~1200 °C and kept for 4 h, then cooled with the furnace, and the white powder obtained is the required BN nanometer material.

### 2.2. Preparation of BN Nanotubes

Mix boric acid, urea, and catalyst ferric nitrate in a certain proportion (1:4:0.01~0.16 molar ratio) in the glove box, put the powder into a covered crucible and put it into a tubular atmosphere furnace. Raise the temperature to 950 °C under argon protection atmosphere, and then inject ammonia, raise the temperature to 1000~1500 °C and keep it for 4 h, and then cool down with the furnace.

The obtained powder is dispersed in dilute hydrochloric acid solution and magnetically stirred for 12 h, the precipitation is transferred to dilute nitric acid solution and stirred for 2 h, and the required BN nanotube material is obtained after filtration, washing, and drying.

### 2.3. Preparation of BN Nano-Material Composite Fluorescent Film

Add the prepared BN nanomaterial into the PVP-acetone-ethanol (1:0.05 mass ratio) mixed solution for ultrasonic dispersion for about 5 h, then add a certain amount of phosphor and glass powder and mix them evenly through a ball mill to dry the obtained mixture, and finally prepare the BN nanomaterial composite PiG fluorescent film according to the steps in the previous work [20].

### 2.4. Characterization

The crystal structure and phase formation were analysed by X-ray powder diffraction (XRD) with Cu-Ka radiation (D/max 2550 PC Rigaku, λ = 0.10405 nm). The Raman spectra were obtained by confocal micro-Raman spectrometer (Renishaw InViaReflex, Gloucestershire, UK; 785 nm). The morphology of the samples was inspected using a scanning electron microscope (Hitachi SEM S-3000, Tokyo, Japan) and transmission electron microscope (Hitachi TEM H-800, Tokyo, Japan). The PL properties were measured using a fluorescent spectrophotometer (JASCO FP-6600, Tokyo, Japan). The high-temperature stability of BN nano-materials was tested using the Discovery TGA thermal analysis system (TA Instruments Q5000IR, New Castle, PA, USA; N_2_ or O_2_ is used as the test atmosphere (flow rate 20 mL/min), the temperature rises to 900 °C at 20 °C /min, and then changes to 10 °C /min to 1150 °C). The electronic universal material testing machine (Instron-5969, Buckinghamshire, UK; Three-point bending) was used to test the PiG samples (12 mm × 18 mm × 1.5 mm) with different components (marked with BN rations) to obtain the mechanical strength. The temperatures and infrared thermal images were recorded using an infrared thermometer (FLIR ThermoVision A40M, Wilsonville, OR, USA).

## 3. Results and Discussion

### 3.1. Phase Analysis and Morphology Control of BN Nanomaterials

#### 3.1.1. Phase Analysis

**Figure 1:**
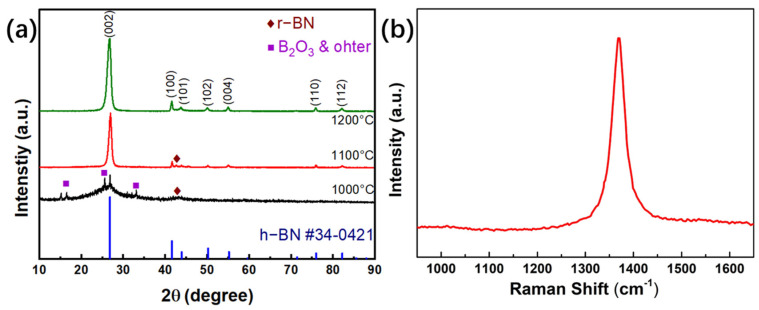
(**a**) XRD of the BN nanomaterial prepared at different temperature; (**b**) Raman spectrum of BN prepared at 1200 °C.

In order to study whether the prepared products are the required boron nitride nanomaterials, XRD tests were carried out for phase analysis. As shown in Figure 1a, when the synthesis temperature is 1000 °C, the products are mainly composed of boron oxides and amorphous substances, with only a small amount of hexagonal boron nitride (h-BN) and cubic boron nitride (r−BN). When the temperature rises to 1100 °C, h-BN becomes the main crystalline phase, while there is a small amount of r-BN phase. When the synthesis temperature continues to rise to 1200 °C, the r-BN phase has basically disappeared, showing exactly the required h-BN phase. This shows that, in the process of calcination, it is necessary to raise the holding temperature above 1200 °C to basically ensure the synthesis of hexagonal boron nitride.

The graph shows that the strongest characteristic peak at a 2 angle of about 26 degrees corresponds to the (002) crystal plane of h-BN, which is consistent with the phase parameters of BN reported in many previous studies [21].

Figure 1b shows the Raman spectrum of the product obtained at 1200 °C. There is a sharp characteristic peak at 1368 cm^−1^, which corresponds to the E2g in-plane stretching vibration mode caused by the displacement of hexagonal boron nitride B and N atoms, further proving that the product belongs to h-BN phase [22].

#### 3.1.2. Morphology Control

In order to study the effect of different preparation conditions on the morphology of boron nitride nanomaterials, a series of samples were prepared by adjusting the proportion of raw materials, synthesis temperature, and holding time, hoping to obtain BN nanomaterials with various morphologies, such as BNNT, BNNS, and BNNP, in order to explore the effect of their addition on the performance of PiG.

**Figure 2:**
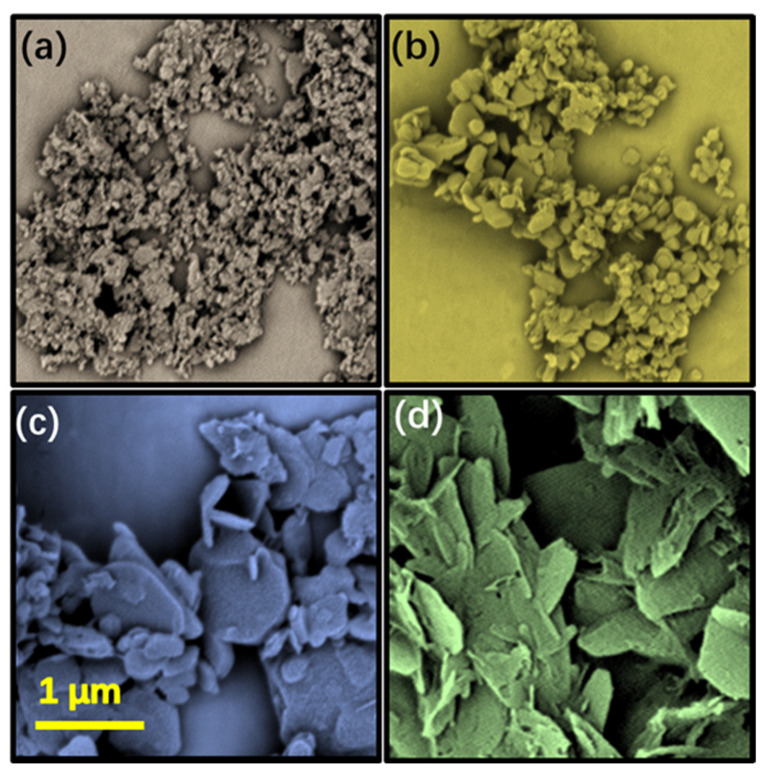
SEM images of BN synthesized with different molar ration of boric acid to ureal (**a**–**d** were 1:2, 1:4, 1:8 and 1:16, respectively).

Figure 2a–d shows the SEM photos of BN nanomaterials obtained when the reaction raw material is free of iron nitrate and the ratio of boric acid to urea is 1:2, 1:4, 1:8 and 1:16. It can be seen from the figure that when the ratio of boric acid to urea is 1:2, most of the products are small nanoparticles with a particle size of about tens of nanometers. When the ratio increases to 1:4, the particle size increases and the morphology begins to flatten. With the further increase of the proportion of urea, the size of BN continues to increase to hundreds of nanometers or even close to 1 micron, and the shape of BN also basically becomes a thicker sheet material. When the ratio of boric acid to urea reaches 1:16, the product has become a nano-layer with a size of more than 1 micron and a thickness of several or dozens of nanometers, which can basically belong to BNNs. To understand the reason for this phenomenon, we need to start with the chemical reaction during the formation of boron nitride. The following reactions mainly occur in the reaction mixture of the crucible as we speculated [23,24]:

2B(OH)_3_ → B_2_O_3_+3H_2_O↑ (1)

B_2_O_3_ → 2B*+3O* (2)

NH_2_CONH_2_ → NH_3_↑+HNCO (3)

2NH_3_ → N_2_↑+3H_2_↑ (4)

N_2_ → 2N* (5)

B*+ N* → BN(6)

**Figure 3:**
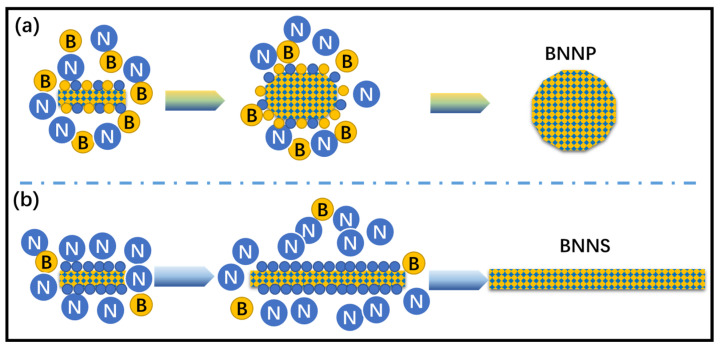
Proposed formation mechanism and schematic illustration of the effect of molar ratio of B to N on the structure growth of BNNP (**a**) and BNNS (**b**).

The speculated forming mechanism is shown in Figure 3a: when the proportion of urea is low, the activated nitrogen atom N* can basically match and combine with the boron precursor B*, and finally successfully synthesize nanoparticles; When the proportion of urea increases significantly, the reactions (3)–(5) in Formula (1) occur intensively and produce a large amount of N*. According to the Curie–Ulf theorem, a large amount of N* will be adsorbed on the (002) crystal surface with high surface energy or adsorption energy, which hinders the continuous growth in this direction, making the crystal tend to grow laterally, and finally forming a larger size and smaller thickness lamellar structure (as shown in Figure 3b).

From Figure 2, it can be found that when iron nitrate is not added to the reaction raw material, boron nitride nanotubes cannot be synthesized, although nano-materials with different morphologies, such as particles and flakes, can be obtained by adjusting the proportion of boron and nitrogen. Compared with nanoparticles, nanotubes have better theoretical properties in terms of heat conduction and mechanical reinforcement, so it is of great significance to obtain BNNT simply and efficiently for the enhancement of PiG.

Researchers found that some transition metal elements have a certain catalytic role in the preparation of boron nitride nanotubes [25]. Therefore, we hope to successfully prepare BNNT and explore the effect of the catalyst on the morphology of nanotubes by introducing different amounts of iron nitrate as catalyst in the preparation process.

**Figure 4:**
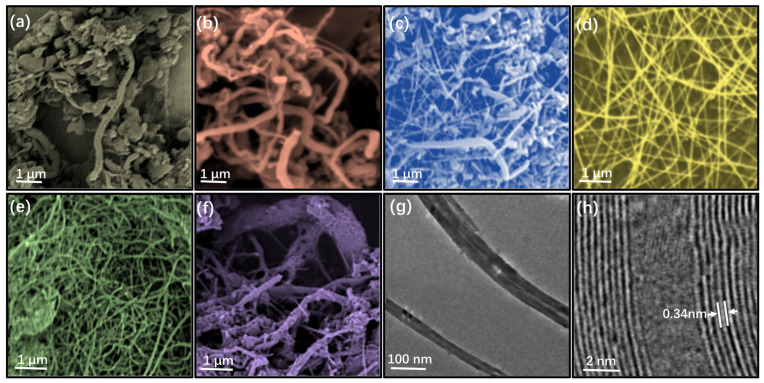
(**a**–**f**) were SEM images of BNNT synthesized with different molar content of Fe (**a**–**f** were1%, 2%, 3%, 4%, 5% and 6%, respectively); (**g**,**h**) were TEM and HRTEM images of BNNT with Fe content of 4%.

Figure 4 shows the SEM photos of the products when the molar content of iron nitrate (relative to boric acid) is 1%, 2%, 3%, 4%, 5%, and 6%. When the content of Fe is 1%, the products are mainly boron nitride nanoparticles, and there are some large nanotubes. With the increase of Fe content, the nanoparticles gradually disappear, and the products are mainly boron nitride nanotubes with large diameter and distorted shape (as shown in Figure 4b). When the amount of catalyst is further increased, nanotubes (4c) with smaller diameter and longer shape appear in the sample, and gradually become the main product. From the SEM, TEM, and HRTEM of the product when the proportion of Fe shown in Figure 4d,g,h is 4%, it can be found that the diameter and length of nanotubes are in the range of tens of nanometers and tens of microns, the tube wall is composed of multilayer boron nitride single wall, and the tube spacing is about 0.34 nm. When the content of Fe increases to 5%, the diameter of the nanotube becomes larger again and the shape changes from the long straight state to the twisted state, and continues to grow with the increase of the content of Fe. Finally, when the proportion of catalyst increases to 6%, various irregular forms appear.

Based on the above phenomena, the relevant formation mechanism is speculated, and the chemical reaction Formula (2) is used to explain the cause of this phenomenon:

2B(OH)_3_ → B_2_O_3_+3H_2_O↑ (7)

4Fe(NO_3_)_3_ → 2Fe_2_O_3_ + 4NO_2_↑ + 11O_2_↑ (8)

NH_2_CONH_2_ → NH_3_↑+HNCO (9)

B_2_O_3_ +3Fe_2_O_3_+8NH_3_ → 2B*+2N* +6Fe +12H_2_O↑+3N_2_↑ (10)

Fe_2_O_3_+ 2NH_3_ → 2Fe +3H_2_O↑+N_2_↑ (11)

B*+ N* → BN(12)

The presence of Fe in the raw material makes the reaction of formula (10) exist in the synthesis process of boron nitride. Compared with the direct decomposition of B_2_O_3_ and N_2_ in Formulas (2) and (5) to generate activated boron B* and nitrogen N*, the activation energy of this reaction formula is lower, so B* and N* can be rapidly generated, and BNNT can be generated under the catalysis of Fe core [23]. When the content of Fe is low, the number of Fe cores is also low, so the number of nanotubes that can be generated is small, so the larger tube body is generated under the stacking of B* and N*. When Fe content increases, BNNT with large number but small size can be synthesized due to the increase in the number of Fe cores.

However, when the content of Fe is further increased, NH_3_ and Fe_2_O_3_ tend to react to (11) in the presence of a large amount, which will hinder the reaction (10); At the same time, in the absence of B * and N * around, a large number of Fe atoms will gather to form Fe simple substance, which will eventually hinder the formation of BNNT and destroy the morphology of nanotubes.

**Figure 5:**
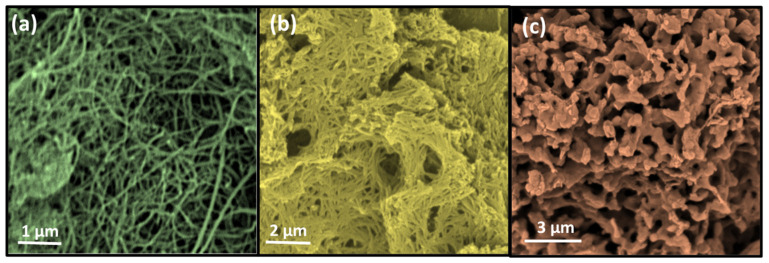
SEM images of BNNT synthesized at 1200 °C (**a**), 1350 °C (**b**) and 1500 °C (**c**).

The synthesis of boron nitride nanotubes is a comprehensive reaction under high temperature environment, so it is speculated that the synthesis temperature may have a great impact on the product morphology. Figure 5 shows SEM photos at synthetic temperatures of 1200 °C, 1350 °C, and 1500 °C. It can be seen from the figure that when the temperature is 1200 °C, which is the preparation temperature of the previous series of samples, the products are mainly nanotubes with good morphology. However, when the temperature rises to 1350 °C, the nanotubes begin to agglomerate and adhere. This is because the surface of boron nitride nanotubes can be corroded and damaged by some Fe-B substances at higher temperatures. At the same time, higher temperatures will cause some activated atoms to adhere to the tube wall and adhere to each other before forming nanotubes. When the synthesis temperature changes to 1500 °C, there is basically no nanotube structure in the product, and only some interconnected particles form a hole structure. This may be because BNNT only forms the initial tubular structure and skeleton, but is surrounded by a huge number of active atoms, such as B* and N*, before it grows up, and finally forms the structure in Figure 5c.

### 3.2. Stability and Distribution Properties of BN Nanomaterials

#### 3.2.1. High Temperature Stability of BN

During the preparation of boron nitride nanomaterial-PiG composite sheet, it needs to go through a period of high temperature heating, so the physical and chemical stability of BN in high temperature environment is crucial.

**Figure 6:**
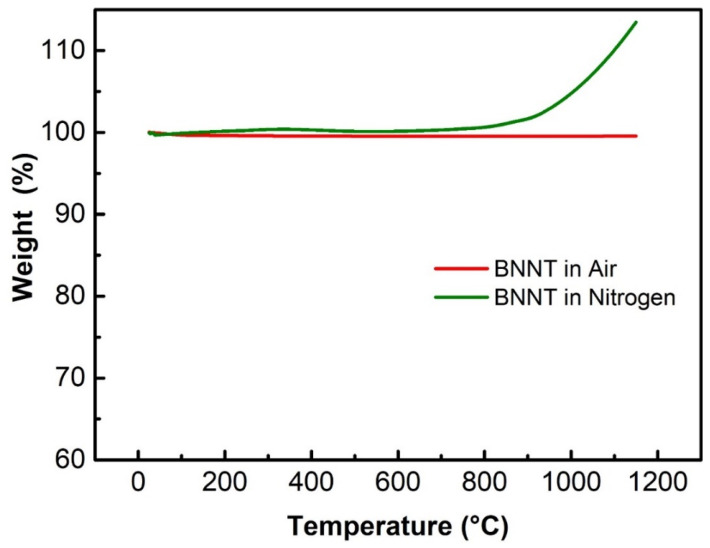
TG curves of the as-prepared BNNT in air and nitrogen.

Figure 6 shows the thermogravimetric curve of boron nitride nanotubes in air and nitrogen protection atmosphere. It can be found that BNNT is extremely stable in nitrogen environment. Except for the loss of a small amount of free water and impurities adsorbed on the surface, the weight of BNNT is basically unchanged; In the air environment, only surface oxygen adsorption-desorption and nozzle BN oxidation caused a little mass change in the early stage. Only when the temperature exceeded 800 °C did the mass of BNNT begin to change significantly [6]. This shows that boron nitride nanotubes are stable under 800 °C in nitrogen or air. The preparation process of composite sheet is basically conducted below 600 °C, and the self-made glass liquid does not contain alkali metal or other elements that can destroy BN, so BNNT can maintain good physical and chemical properties.

#### 3.2.2. Distribution of BN Nanomaterials

BN nano-materials prepared in the early stage are generally small in size and easy to agglomerate, which is not conducive to the dispersion and performance of the materials added into PiG in the later stage. Therefore, in the preparation process of composite phosphor, PVP was used to disperse boron nitride nano-materials, and the phosphor and glass powder were fully mixed by ball milling, hoping to obtain a PiG layer with relatively uniform BN distribution.

**Figure 7:**
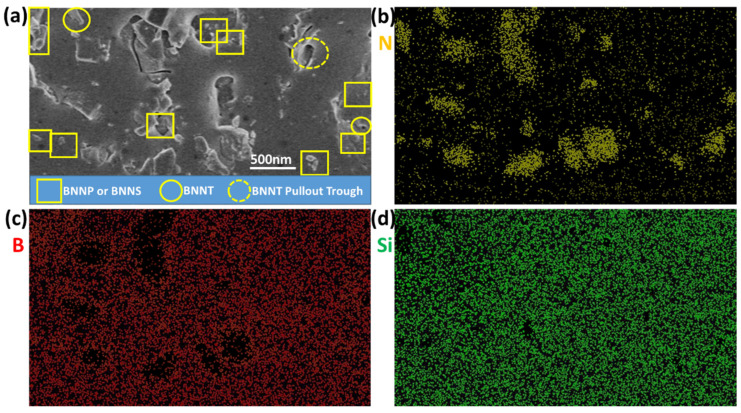
SEM image (**a**) and eds mapping (**b**–**d** were N, B and Si, respectively) of cross-section of BN/PiG composite.

Figure 7 shows the SEM photos and element distribution of the fracture surface of BN nano-material composite fluorescent film. From the SEM photos, it can be found that because the size of BN is too small, it is necessary to rely on the element distribution to help judge the true composition. In the three main components of BN-PiG composite system, containing phosphor, glass phase, and boron nitride, phosphor and glass have Si element when they are the same, phosphor and boron nitride have N element at the same time, while boron nitride and glass phase have B element, so the test and analysis of these three elements are mainly carried out. When there are B and N elements in the material in a certain region and the distribution of Si is lacking, it can be basically identified as boron nitride nanomaterials. Therefore, the distribution of boron nitride nano-materials in the photos was judged and confirmed by combining SEM and element distribution: boron nitride nano-sheets, nano-particles, and nanotubes were evenly distributed in the thin slices, without large agglomeration. The size of the nanotubes and the grooves left after pulling out are relatively consistent, indicating that the nanotubes are closely connected with the solid phase. This has laid a good foundation for our next research.

### 3.3. Effect of BN Nano-Materials on the Mechanical Properties of Fluorescent Films

Boron nitride nanomaterials are similar to carbon materials, which have planar six-element crystal structure and strong mechanical properties and are often used as reinforcing phases of ceramic materials.

As a pure inorganic material formed by the combination of ceramic phase phosphor and glass, PiG will have insufficient mechanical properties when it is relatively thin, and will be easily damaged during preparation, transportation or use, thus losing its application value. Therefore, adding boron nitride nano-materials into PiG can enhance the mechanical strength of the sheet layer, allowing to explore the effect of different boron nitride content and morphology on the performance.

**Figure 8:**
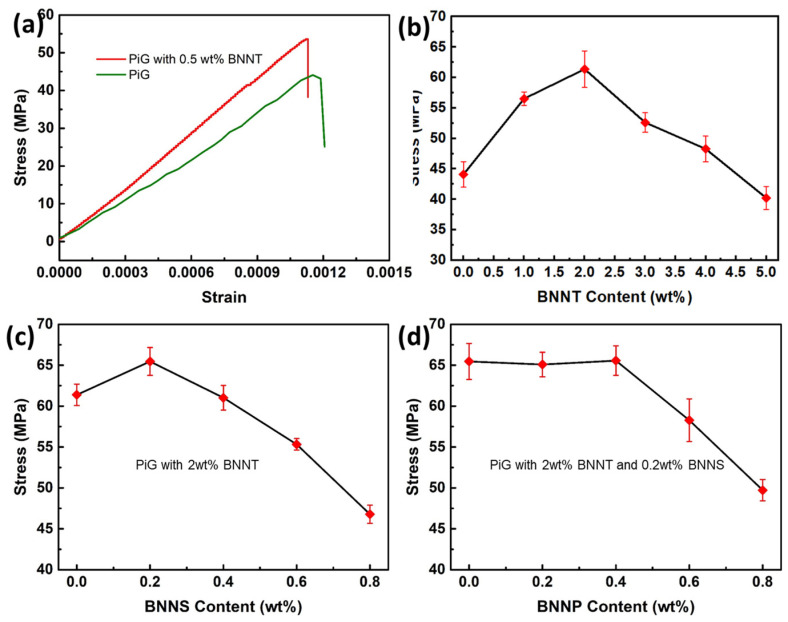
(**a**) Stress-strain curves of PiG and the one with 0.5 wt% BNNT; (**b**–**d**) Flexural strength of PiGs with different content of BNNT, BNNS and BNNP.

Figure 8 shows the effect of the addition of boron nitride nanomaterials on the mechanical properties of PiG. The stress–strain curve of the sheet was measured by three-point bending method and its bending strength was obtained. When BNNT with different mass fractions (relative to the sum of the mass of phosphor and glass powder) is added to PiG, the bending strength of the sheet presents a trend of first increasing and then decreasing (Figure 8b): when the addition ratio is 2 wt%, the bending strength reaches approximately 61 MPa at the highest, which is about 39% higher than that of PiG without BNNT. However, when the content of BNNT is further increased, the bending strength decreases rapidly. It is speculated that the increase of the content may cause the nanotubes to agglomerate to a certain extent, or that the excessive number of nanotubes hinders the adhesion of the glass liquid to the phosphor particles.

In material mechanics, fillers with different morphologies generally play a complementary role in performance [26]. Therefore, after adding a certain amount of BNNT, we also added different amounts of BNNS and BNNP, hoping to obtain the best addition ratio and best mechanical properties. Based on adding 2 wt% BNNT, when the content of BNNS is 0.2 wt%, the strength reaches the maximum value of about 65 MPa, with an increase of approximately 6.5%, indicating that the addition of nanoflakes can also play a certain role in enhancing PiG (Figure 8c). Then, on the basis of adding 2 wt% BNNT and 0.2 wt% BNNS, we continued to add different contents of BNNP. It was found that when the content was low, the performance could not be affected basically. When the content was 0.4 wt%, it only increased by less than 1%, and when the content was high, it would significantly reduce the strength, indicating that the nanoparticles could not be further improved based on the strengthened nanotubes and nanoflakes.

### 3.4. Effect of BN Nano-Materials on Heat Dissipation Performance of Fluorescent Fin

Theoretically, the higher the content of boron nitride in the sample composition, the better the thermal conductivity and heat dissipation of the sheet. However, the increase of its content will inevitably affect the overall fluorescence and mechanical properties of the sheet, and may even lead to the inability to prepare PiG sheets. In terms of mechanical property enhancement, the addition of boron nitride nano-materials with different morphologies has an impact on the flexural strength of PiG and has a certain supplementary effect. Therefore, when studying the enhancement effect of boron nitride nano-materials on the heat dissipation performance of phosphors, we added different forms of BN materials to explore the effect of material morphology on the performance.

**Figure 9:**
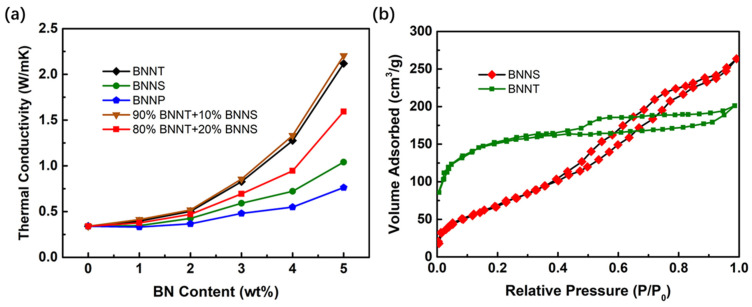
(**a**)Effect of BN nanomaterials with different morphology on the thermal conductivity of PiG; (**b**) Nitrogen adsorption–desorption isotherm of BNNT and BNNS.

Figure 9a shows the change of thermal conductivity of PiG sheets with different forms of boron nitride nanomaterials. It can be found that with the increase of the mass ratio of boron nitride, the thermal conductivity keeps rising, but the rate of rise is different. Among BNNT, BNNS, and BNNP, BNNT obviously contributes more to the thermal conductivity than the other two, because the shape of the filler has a great impact on the thermal conductivity of the composite. Fillers with a certain length-diameter ratio, such as fibrous, needle-like, and rod-like, are able to form a thermal conductivity network more easily when added to the matrix, thus improving the thermal conductivity of the material. The BET test was adopted to obtain the surface to volume ratios of BNNT and BNNS, as shown in Figure 9b. The higher specific surface area of BNNT (503.6 m^2^/g) than BNNS (265.3 m^2^/g) signifies larger heat-exchange area between BNNT and PiG material. The increased heat exchange, working together with the strong axial heat conduction ability of nanotubes, contributes to the improved thermal performance of PiG with BNNT.

However, nanotubes and other materials also have certain disadvantages, that is, they cannot be fully and uniformly distributed like nano-sheets and nanoparticles, which will also affect the overall thermal conductivity. Therefore, we mixed 10% nano-sheet material into boron nitride nanotubes, and checked its thermal conductivity. It was found that its thermal conductivity was improved compared with the single nanotube material, probably because a small amount of nano-sheet helped improve its thermal conductivity network. However, when the proportion of nano-sheets is further increased to 20%, the thermal conductivity decreases significantly, which may be due to the relatively large damage to the thermal conductivity network with the reduction of nanotubes.

**Figure 10:**
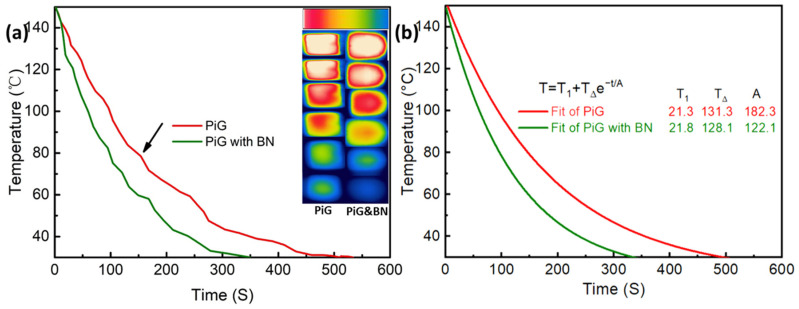
The cooling curves (**a**), fitted curves (**b**) and Infrared thermal images (insert in a) of PiG and PiG with BN.

In order to directly observe the enhancement effect of boron nitride nano-materials on the heat dissipation ability of PiG, a cooling test was also taken to characterize its heat dissipation in the air environment. Figure 10a shows the temperature drop curve of PiG and PiG added with boron nitride nanomaterials (total mass fraction of 5 wt% BNNT and BNNS, of which BNNT accounts for 90%) from 150 °C to 30 °C at room temperature. From the curve, it can be found that after adding boron nitride nanomaterials, the cooling rate of PiG was improved to a certain extent, and the infrared imaging photos also directly show this result.

According to Newton’s cooling law, the two cooling curves are fitted using Equation (4). As shown in Figure 10b, through fitting, it is found that the comprehensive cooling parameters A of PiG and PiG with BN are 182.3 and 122.1, respectively, which indicates that the introduction of boron nitride nanomaterials such as BNNT and BNNS can effectively reduce the cooling parameters and increase the heat dissipation capacity of PiG.

### 3.5. Effect of BN Nano-Materials on PiG Fluorescence and Packaging Performance

The addition of boron nitride nano-materials effectively enhances the mechanical properties and heat dissipation properties of PiG, but it is bound to affect the luminescent properties of the phosphor. In order to study the change of luminescence properties of PiG after BN addition, and to explore the effect of BN addition amount and morphology on it, we carried out fluorescence spectrum analysis on a series of samples.

**Figure 11:**
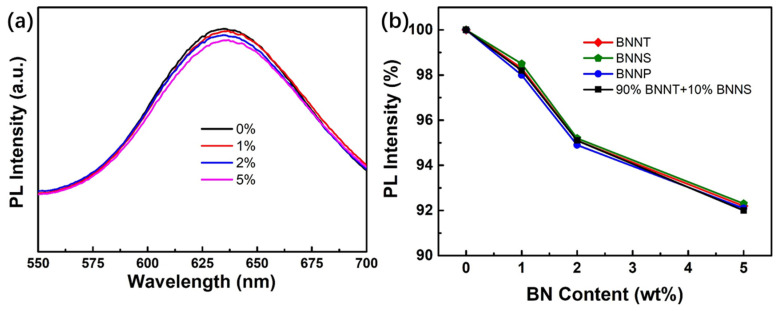
(**a**) Emission spectra of PiG with different content of BNNT; (**b**)PL intensity of PiG with differnent BN nanomaterial.

As shown in Figure 11a, when the mass fraction of BNNT added is 1%, the intensity of the emission spectrum only slightly decreases. When the amount of BNNT continues to increase, the decrease of luminous intensity becomes more obvious. When the amount of BNNT reaches 5 wt%, the intensity decreases by about 2%. It can be predicted that the luminous intensity will continue to decrease when the addition amount continues to increase, mainly due to the relative decrease of phosphor content and the impact of BN nanomaterials on the optical path (although BN materials do not absorb visible light, it is still possible to hinder the light transmission due to scattering and other reasons). In addition, it can be seen from the figure that the shape of the emission spectrum has basically not changed, indicating that the addition of BN will not affect the optical parameters such as the emission wavelength and color coordinates. Figure 11b shows the change of the luminous intensity of PiG with different morphologies of boron nitride nanomaterials. From the figure, it can be found that the decrease of the luminous intensity caused by nanotubes, nanosheets, nanoparticles, and mixtures is basically the same under the same amount of addition, indicating that the morphologies of BN nanomaterials do not have significantly different effects on the luminous properties of PiG.

**Figure 12:**
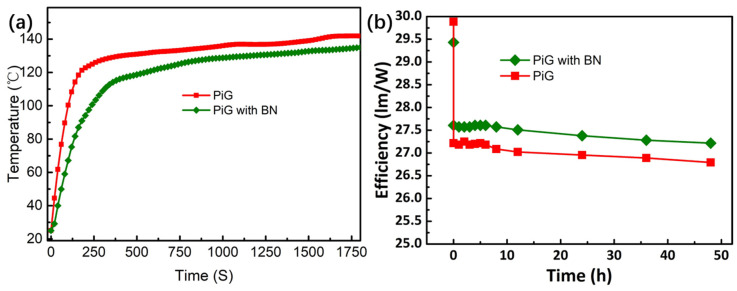
The working-temperature curves (**a**) and luminous efficiency variation (**b**) of LED lamps made of PiG and PiG with BN.

At the same time, referring to the previous packaging method, the PiG with appropriate area is directly placed at a certain distance above the high-power chip to form a simple remote packaging high-power LED. Test the temperature and lumen efficiency of the LED obtained by PiG and PiG packaging with BN, and the results are shown in Figure 12. When the LED starts to work, its temperature rises slowly in the first few seconds, and then rises rapidly due to the large amount of heat release due to the reduction of the conversion efficiency of the fluorescent material or the overall lumen efficiency of the device, and finally maintains in a stable range due to the thermal balance. Compared with PiG without BN, PiG with BN nano-materials for heat dissipation enhancement lags in the initial lumen efficiency, but has higher efficiency in the subsequent time because of the lower operating temperature. This shows that BN nano-materials have successfully improved the luminous performance of the phosphors for high-power LED remote packaging.

## 4. Conclusions

By adjusting the ratio of boric acid to urea in the raw material, boron nitride nanoparticles and nano-sheets with different morphology and size were obtained, which may be due to the influence of the different amount of N * on the growth rate of boron nitride in different directions. With the control of the number of catalysts and the synthesis temperature, a series of nanotubes with different morphologies were obtained: with the increase of the number of iron catalysts, the morphology of nanotubes changed from coarse distortion to thin and smooth, and then formed a broken structure; too high synthesis temperature will also destroy the pipe wall structure. By adding appropriate BN nano-materials, the mechanical strength, heat dissipation performance, and luminous performance of PiG in the high-power LED can be enhanced. SEM and energy spectrum results show that boron nitride nano-materials have a good combination with the lamellae, and nano-materials with different morphologies can play a better role in strengthening the mechanical properties through comprehensive action, and their bending strength can be improved by about 50% at most. Similarly, boron nitride BNNT and BNNS with different morphologies can effectively improve the heat dissipation performance of the phosphor by forming a heat conduction network. The test shows that the comprehensive cooling parameters of PiG and PiG with BN are 182.3 and 122.1, respectively. This makes the phosphor have lower working temperature and higher lumen efficiency in high-power working environments.

## Data Availability

The research data will be available upon request.
